# A Persistent Primitive Hypoglossal Artery As the Sole Supply to the Brain Associated with a Basilar Bifurcation Aneurysm

**DOI:** 10.3389/fneur.2017.00168

**Published:** 2017-04-26

**Authors:** Ming Wang, Jun Gu, Ping Lan, Shu Wan, Yongqing Zhou, Xiujue Zheng, Renya Zhan

**Affiliations:** ^1^Department of Neurosurgery, The First Affiliated Hospital, College of Medicine, Zhejiang University, Hangzhou, Zhejiang, China

**Keywords:** persistent primitive hypoglossal artery, basilar bifurcation, aneurysm, clip, intraoperative neurophysiologic monitoring

## Abstract

The persistent primitive hypoglossal artery (PPHA) is the second most common persistent carotid–vertebrobasilar anastomosis, with an incidence of 0.027–0.26%. PPHAs change the hemodynamics of the carotid and vertebrobasilar system and may be associated with intracranial vascular anomalies, but basilar bifurcation aneurysms were rarely reported. We describe the first case of a PPHA as the sole supply to the brain associated with a basilar bifurcation aneurysm and review the literature. We reported a 34-year-old woman who presented with subarachnoid hemorrhage due to a ruptured basilar bifurcation aneurysm. Digital subtraction arteriogram revealed a right PPHA as the sole supply to the brain. The aneurysm was successfully clipped under intraoperative neurophysiology.

## Introduction

The persistent primitive hypoglossal artery (PPHA) is the second most common persistent carotid–vertebrobasilar anastomosis, with an incidence of 0.027–0.26% (1). PPHAs change the hemodynamics of the carotid and vertebrobasilar system and may be associated with intracranial vascular anomalies. The incidence of concomitant intracranial aneurysms and PPHA was about 26% (2), but basilar bifurcation aneurysms were rarely reported. To date, only four cases of PPHA associated with basilar bifurcation aneurysms have been reported in English in the literature (3–5). In this article, we describe the first case of a PPHA as the sole supply to the brain associated with a basilar bifurcation aneurysm, which was successfully clipped under the monitoring of the intraoperative neurophysiology.

## Case Presentation

**Figure 1:**
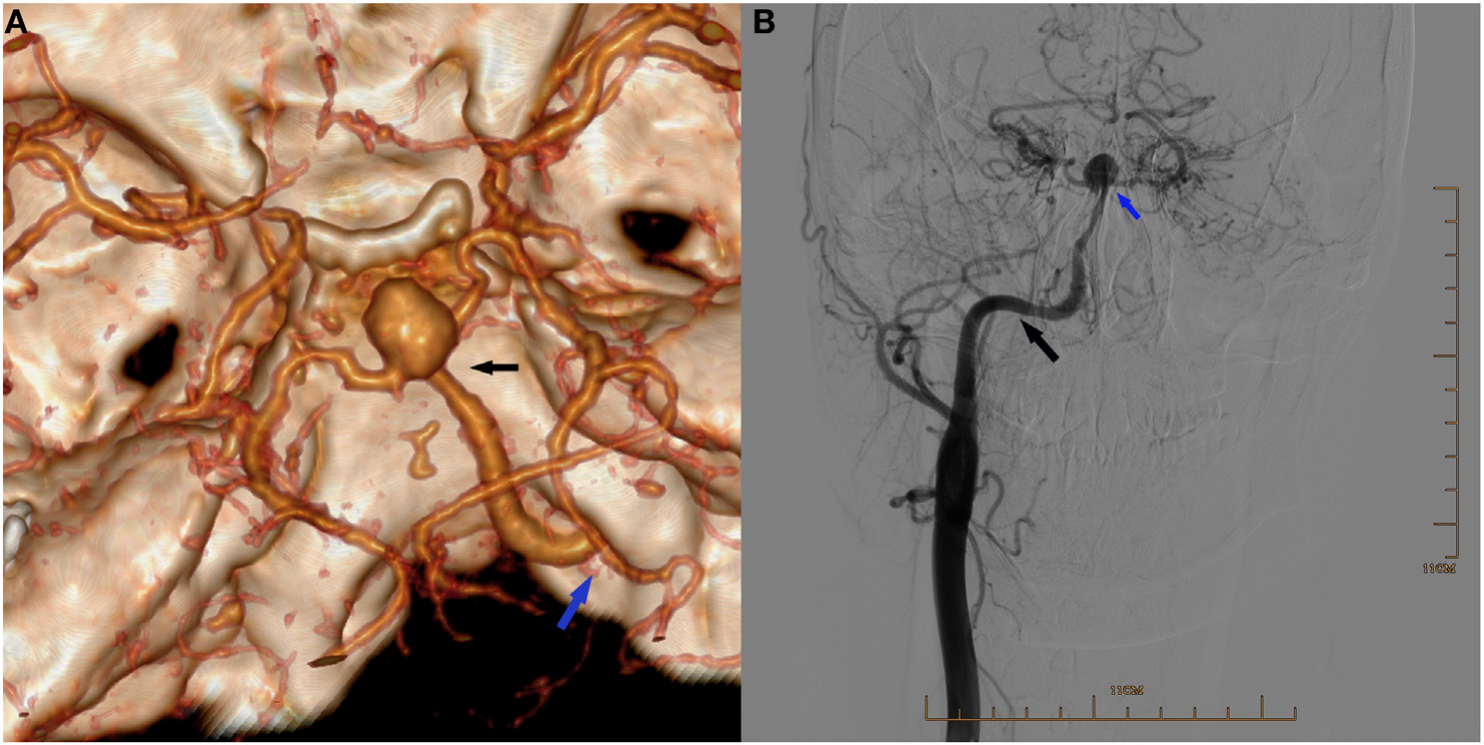
**(A)** Three-dimensional reconstruction of computed tomography angiography showing an aneurysm located at the bifurcation of the right persistent primitive hypoglossal artery (PPHA) and two posterior cerebral arteries (black arrow), and the right PPHA entering the skull *via* the right hypoglossal canal (blue arrow) and supplying the circle of Willis. **(B)** Digital subtraction arteriogram of the right common carotid artery (CCA) showing the PPHA (black arrow) originating from the CCA and an aneurysm in the apex (blue arrow). The right internal carotid artery was absent.

**Figure 2:**
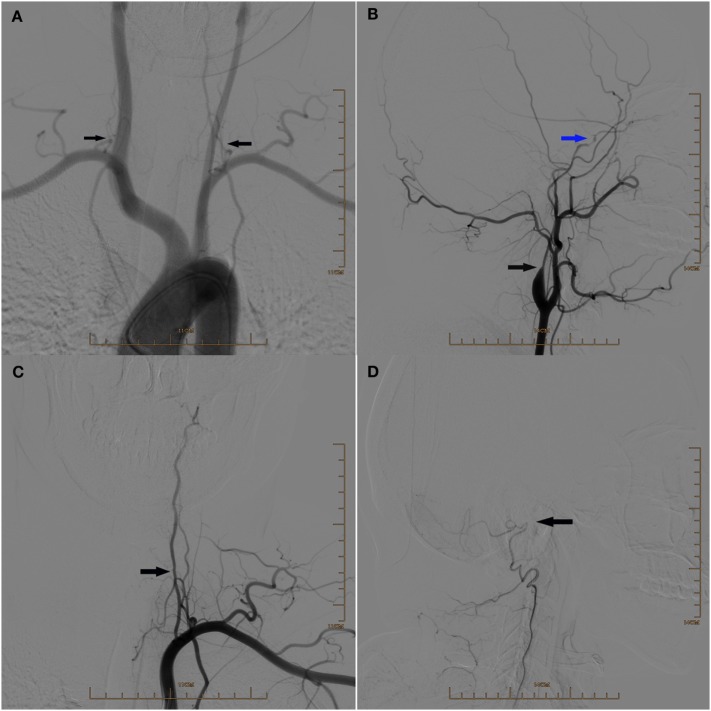
**(A)** Digital subtraction arteriogram (DSA) of the aortic arch showing the hypoplasia of both vertebral arteries (arrows). **(B)** Lateral view of DSA of the left common carotid artery showing the hypoplasia of the left internal carotid artery at the initial segment (black arrow) and occlusion above the beginning of the left ophthalmic artery (blue arrow). **(C)** DSA of the left subclavian artery showing the hypoplasia of the left vertebral artery (arrow). **(D)** Lateral view of DSA showing the occlusion of the left vertebral artery above the beginning of posterior meningeal artery (arrow).

**Figure 3:**
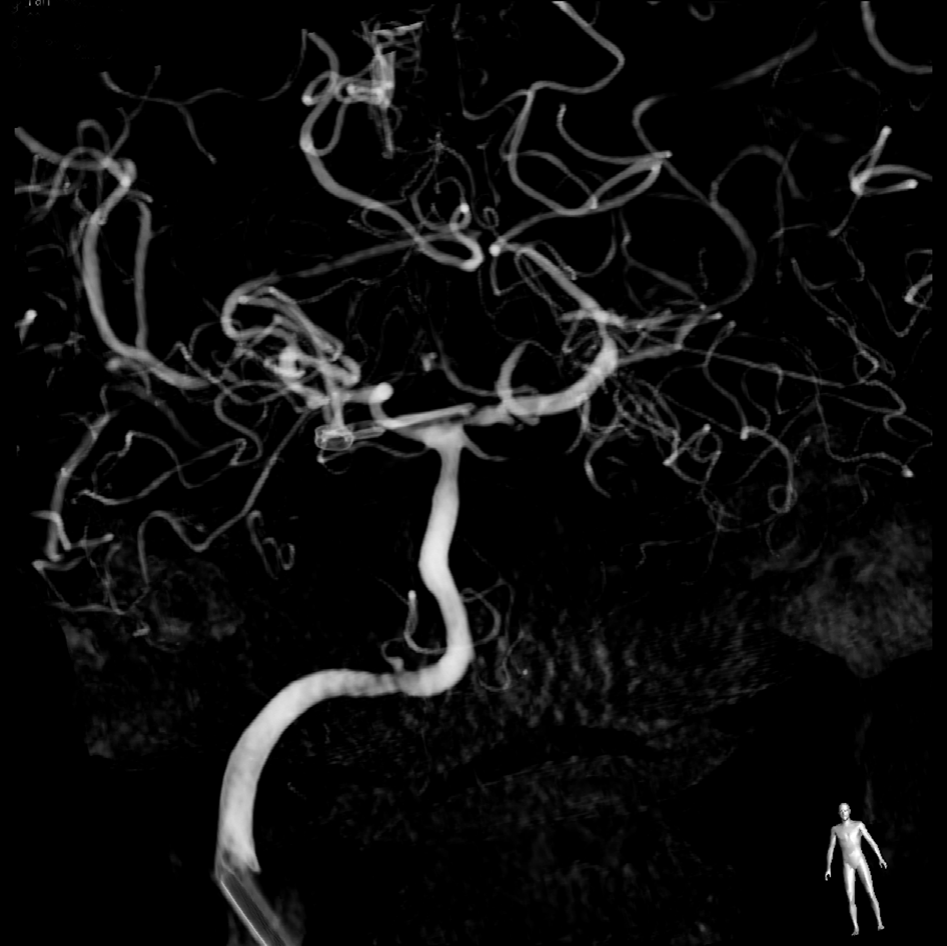
**Digital subtraction arteriogram of the right common carotid artery showing the aneurysm was completely clipped**.

A 34-year-old woman presented with sudden headache associated with nausea and vomiting. She had a history of hypertension for 10 years. Nothing but positive cervical resistance was found by neurological examination. Computed tomography (CT) indicated subarachnoid hemorrhage. Echocardiography indicated congenital heart disease of primary pass atrial septal defect with pulmonary hypertension. CT angiography and digital subtraction arteriogram revealed a broad-neck basilar bifurcation aneurysm and the presence of a PPHA originating from the right common carotid artery, turning posterior, entering the skull through the hypoglossal canal, and extending as the basilar artery (Figure 1). Both vertebral arteries were hypoplastic. The right internal carotid artery (ICA) was absent, while the left ICA was occlusive above the beginning of the left ophthalmic artery (Figure 2). The anterior circulation was supplied by the posterior circulation *via* posterior choroidal arteries. Therefore, the right PPHA was the sole supplying artery to the brain. Clipping of the aneurysm was performed under the monitoring of the intraoperative neurophysiology, including bilateral somatosensory evoked potentials (SSEPs) and motor-evoked potentials (MEPs), with routine intravenous–inhalation combined anesthesia, keeping the mean arterial pressure (MAP) of 50–60 mmHg. During the operation, bilateral SSEPs and MEPs started to descend, 3 min after temporary clipping basilar artery. We stopped the clipping immediately and raised the MAP to 70–75 mm HG. SSEPs and MEPs recovered, 15 min later. Finally, the aneurysm was clipped successfully (Figure 3). Postoperatively, the patient presented with apathy and muscle weakness, and CT revealed infarction in left frontal lobe, which may due to vasospasm caused by preoperative serious subarachnoid hemorrhage and temporary clipping basilar artery during the operation. After the treatment of hyperbaric oxygen and rehabilitation of limb function, the patient gradually improved and she was discharged with fine movement deficit of the hands. Follow-up angiography demonstrated the aneurysm was completely clipped, and the patient could engage in light manual labor with normal neurological function three months after the operation with GOS score of 5.

## Discussion

Embryonic carotid–vertebrobasilar anastomosis includes the trigeminal artery, hypoglossal artery, otic artery, and proatlantal artery, which supply the posterior circulation in the early fetal brain. Failure of regression of presegmental arteries will result in the onset of persistent primitive arteries. PPHA represents the second most common persistent carotid–vertebrobasilar anastomosis, after the persistent trigeminal artery, estimated to occur in 0.027–0.26% of the population (1).

Persistent primitive hypoglossal artery generally is an incidental angiographic finding, as it may be completely asymptomatic. Nonetheless, the fatal clinical feature of PPHA is its association with intracranial aneurysms, with an incidence of 26% (2). It is still controversial that PPHA increases the risk of intracranial aneurysms (6), but hemodynamic alteration in the carotid and vertebrobasilar system and association with anomalous structure of the vessel wall predispose to the onset of aneurysms. In our case, the hypoplasia of both vertebral arteries, the absence of the right ICA, and the occlusion of the left ICA make the right PPHA the exclusive feeder of the circle of Willis. Reviewing the published literature, only one definite case of PPHA as the sole supply to the brain was reported (7). More specifically in our case, a basilar bifurcation aneurysm was associated with PPHA. To the best of our knowledge, only four cases of basilar bifurcation aneurysm associated with PPHA, two cases of left PPHA and two cases of right PPHA, have been reported in English before (3–5). The first case of basilar bifurcation aneurysm associated with PPHA was a 29-year-old female, presenting of unconsciousness and a series of epileptic fits. Because of the bad condition, she did not get the treatment of aneurysm and died 39 days after the initial hemorrhage and necropsy found a left persistent hypoglossal artery, with diameter of 3 mm and a basilar bifurcation aneurysm (5). Another case of right persistent hypoglossal artery, accompanied by a basilar bifurcation aneurysm, was reported to be treated by clipping (4). Moreover, Sakai et al. reported two similar cases. The two aneurysms were both completed clipped, and patients got transient left abducens palsy and right oculomotor nerve palsy, respectively (3). Here, we report the first case of a broad-neck basilar bifurcation aneurysm in the setting of a PPHA as the sole supply to the brain.

The management is challenging, either for surgical clipping or for endovascular treatment. Endovascular management could avoid surrounding tissue disruption, but stent-assisted coiling was needed for this the broad-neck aneurysm, which increased the risk of cerebral vasospasm and thrombembolia. Additionally, the malformation of the Willis circle causes lack of collateral circulation, which makes endovascular treatment much more difficult and challenging. So surgical clipping under the monitoring of the intraoperative neurophysiology was chosen for this patient. During operation, 3-min blocking of the proximal basilar artery during clipping resulted in decrease SSEPs and MEPs, which probably was responsible for postoperative cerebral edema and low level of pituitary hormones, which suggested intraoperative neurophysiologic monitoring is necessary to improve the safety of surgical management. Additionally, persistent primitive arteries, as a congenital vascular abnormality, could be associated with general vascular abnormalities. In this case, the patient suffered from congenital heart disease of primary pass atrial septal defect with pulmonary hypertension simultaneously. So it demonstrated that a general examination, especially cardiovascular examination, should be performed before the operation, in order to avoid the omission of serious comorbidities.

## Conclusion

We presented the first case report of a PPHA as the sole supply to the brain associated with a basilar bifurcation aneurysm. The management of PPHA as the sole supply to the brain associated with a basilar bifurcation aneurysm is challenging, either for surgical clipping or for endovascular treatment. Intraoperative neurophysiologic monitoring is necessary to improve the safety of surgical management.

## Informed Consent

This study was carried out in accordance with the recommendations of “Ethics committee of the First Affiliated Hospital, College of Medicine, Zhejiang University” with written informed consent from all subjects. All subjects gave written informed consent in accordance with the Declaration of Helsinki.

## Author Contributions

MW was responsible for writing and operation. JG was responsible for writing and DSA. PL was responsible for intraoperative neurophysiology and data collection. SW was responsible for article preparation, reviewing, and revision. YZ, XZ, and RZ were responsible for operation.

## Conflict of Interest Statement

The research was conducted in the absence of any commercial or financial relationships that could be construed as a potential conflict of interest.

## Abbreviations

CT, computed tomography; CTA, computed tomography angiography; DSA, digital subtraction arteriogram; MEPs, motor-evoked potentials; PPHA, persistent primitive hypoglossal artery; SSEPs, somatosensory evoked potentials.
